# Collimation principles of a hollow X-ray microbeam for high-contrast cytoplasm irradiation

**DOI:** 10.1093/jrr/rrae046

**Published:** 2024-08-18

**Authors:** Qinqin Cheng, Ruifeng Zhao, Xiaowa Wang, Xufei Wang

**Affiliations:** Institute of Modern Physics, Fudan University, Shanghai 200433, China; Key Laboratory of Nuclear Physics and Ion-beam Application (MOE), Fudan University, Shanghai 200433, China; Department of Radiation Oncology, Tongji University School of Medicine, Shanghai Pulmonary Hospital, Shanghai 200433, China; Institute of Modern Physics, Fudan University, Shanghai 200433, China; Key Laboratory of Nuclear Physics and Ion-beam Application (MOE), Fudan University, Shanghai 200433, China; Shanghai Proton and Heavy Ion Center, Shanghai 201321, China; Institute of Modern Physics, Fudan University, Shanghai 200433, China; Key Laboratory of Nuclear Physics and Ion-beam Application (MOE), Fudan University, Shanghai 200433, China

**Keywords:** hollow X-ray microbeam, subcellular radiosensitivity, cytoplasm irradiation, Monte Carlo simulation

## Abstract

A Monte Carlo simulation was used to assess the performance of a collimated hollow X-ray microbeam for subcellular cytoplasm irradiation. A high-Z coaxial collimation structure with an inner core for nucleus shielding was investigated. Two key performances, the extraction efficiency (cytoplasm dose per unit incident fluence) and the dose contrast (cytoplasm-to-nucleus dose ratio), were evaluated regarding the influences of the material, geometry and physical arrangements of the collimator, target dish and incident beam source. Simulation results demonstrate that a gold coaxial structure with a practical collimation geometry of a 1-mm length, 10-μm inner diameter and 200-μm outer diameter, with the top exit closely attached (with a minimized air gap) to the bottom of a cell dish with a 3-μm thick Mylar film is recommended for cytoplasm irradiation of adherent mammalian cells. For a synchrotron source in the energy range < 10 keV, a dose contrast of approximately 100 can be achieved. For a bremsstrahlung source <30-kV tube voltage, a dose contrast of approximately 50–100 can still be achieved. General principles are summarized with further explanations of the performance of the hollow X-ray microbeam.

## INTRODUCTION

Subcellular radiosensitivity is a fundamental but still open issue in radiation biology because of the inability to strictly measure the contribution weight of subcellular domains or components in radiation-induced cell killing. Microbeam technology has been widely used in radiobiology studies [1–5]; however, much of this technology was developed for high-resolution irradiation of subcellular targets by using a solid beam spot. Consequently, the cytoplasm surrounding a nucleus could not be selectively exposed to a uniform dose; this makes the most basic weights of the nucleus and cytoplasm in radiation killing difficult to measure. The unknown relationship between the cytoplasmic dose and cell death makes it difficult to predict the effectiveness of high-Z nanoparticle-mediated cell radiosensitization [6], where the local dose-enhanced killing events in a target cell are mostly induced on extranuclear organelles near the cytoplasm-distributed nanoparticles [7].

In this study, a high-Z coaxial collimation structure was investigated for extracting a hollow X-ray microbeam for cytoplasm irradiation of adherent cells. Such a collimated microbeam has been reported by the Photon Factory group for subcellular irradiation use [8, 9]. However, to the best of our knowledge, there is still a lack of general principles that can provide basic guidance for the geometry, material and physical arrangements of the high-Z coaxial structure for microbeam collimation and subcellular irradiation use. Therefore, this study sought to uncover the influencing factors on the microbeam performance and to propose general principles for the collimation and cell irradiation arrangement for technical guidance. By applying Monte Carlo simulations to the collimation geometry and investigating pretarget media and cell dish arrangements and the energy and angular divergences of the incident beam source, two key performances of the hollow X-ray microbeam were evaluated: the extraction efficiency, defined as the average dose extracted to target cytoplasm per unit fluence of the incident beam, and the dose contrast, defined as the cytoplasm to nucleus ratio of dose. A coaxial model of gold was established for the simulation using the Geant-4–based simulation tool TOPAS 3.7.0 [10, 11].

## MATERIALS AND METHODS

### Initial model and simulation settings

**Fig. 1:**
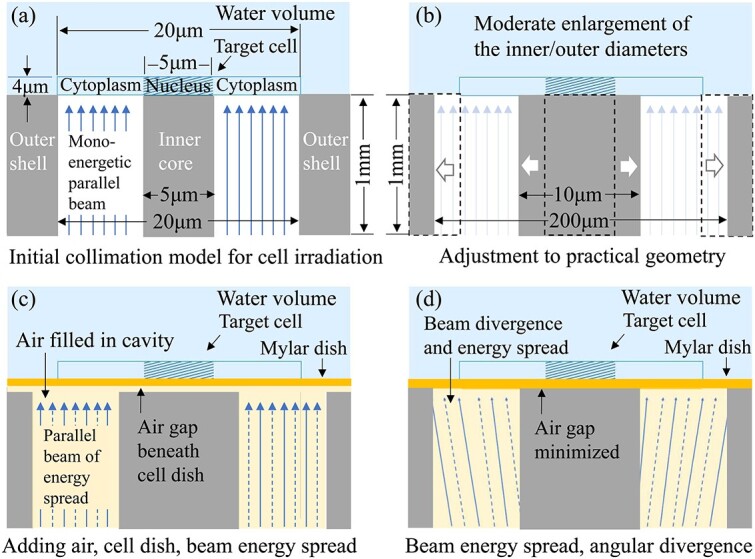
Simulation procedures: (a) an initial model of gold (5-μm inner diameter, 20-μm outer diameter and 1-mm length) for the collimation of a monoenergetic parallel beam; (b) geometry adjustment (diameters and length); (c) adding the pretarget air, cell dish and energy spread of an assumed parallel beam source; (d) evaluation of the combined influence of the energy spread and angular divergence of a bremsstrahlung radiation source.

An initial model (Fig. 1a) for the hollow beam collimation was first established for a basic performance evaluation. At the exit of the collimator, a target cell was modeled as a flat cylinder with a 20-μm diameter and 4-μm thickness, containing a smaller flat cylinder in the center as a nucleus with a 5-μm diameter and the same 4-μm thickness. Importantly, for adherent mammalian cells, the diameters and thickness of an entire target cell and its nucleus can vary depending on factors such as cell type, cell growth condition and the techniques used for size measurement. However, the nucleus and cell diameters are typically 5–10 [12, 13] and 10–30 μm [14], while the average cell thickness is approximately 4 μm [13]. Therefore, for the simplified cell model, the mean sizes and thickness were selected as above based on literature data. The cell was immersed and centered at the bottom of a cylindrical volume of water with a 50-mm diameter and 10-mm thickness (outside the image). Water was used as the equivalent material for the cell model. The model sizes are given in Fig. 1a.

Beneath the water volume containing the cell, gold was selected as the collimator material in consideration of its high-Z quality and easy machining. The coaxial structure had an initial length of 1 mm, with a cylindrical shielding core with a 5-μm diameter in the center, and an external shielding shell with a 20-μm inner diameter and a 20-mm outer diameter (outside the image). The initial 1-mm length can ensure a near complete shielding of low-energy photons of <100 keV, with a transmission ratio of < 0.00005. For extracting an initial hollow spot beam to the target cytoplasm, the inner shielding core was aligned to the target nucleus with the equivalent 5-μm diameter, while the opening hole of the external shielding shell (i.e. the collimator entrance) was aligned to the edge of the cell with the same 20-μm diameter. The 20-mm outer diameter (outside the image) of the shielding shell ensures sufficient shielding of the scattered photons that leak from the side surface to the periphery of the target cell. The initial sizes of the collimator are given in Table 1 (line a) and plotted in Fig. 1a. In the following text, the diameter of the internal shielding core is referred to as the inner diameter of the collimator, while the diameter of the opening hole (the collimator entrance) of the external shielding shell is referred to as the outer diameter of the collimator.

**Table 1:** Simulations on the microbeam performance with regard to (a–e) the collimation geometry, (f–h) the pretarget materials (cell dish, intracavity air and air gap) and (i–k) the incident beam source characteristics

	Collimation geometry			Cell dish	Pre-target air		Beam source		
	OD	ID	Length	Mylar	In cavity	Air gap	Size	Divergence	Energy
(a)	20 μm	5 μm	1 mm	w/o	w/o	w/o	24 μm	Parallel	1–100 keV
(b)	20 μm	5 μm	10 mm				24 μm		
(c)	20 μm	5–18 μm	1 mm				24 μm		
(d)	50 μm	5–25 μm	1 mm				54 μm		
(e)	10 mm	5–25 μm	1 mm				11 mm		
(f)	200 μm	10 μm	1 mm	w/o	w/o	w/o	220 μm	Parallel	1–100 keV
(g)				0–20 μm	w/o	w/o			
(h)				3 μm	w/	1–10 μm			
(i)	200 μm	10 μm	1 mm	3 μm	w/	w/o	220 μm	0–1mrad	1–100 keV
(j) (k)								Parallel	5–100 kV
								0–15 °	5–100 kV

The initial model was established in a vacuum. A supporting dish for target cells was absent between the collimator exit and the water volume; the influence of the cell dish is evaluated in a later section. The incident X-ray source was a monoenergetic parallel beam, with the mono energy of 1–100 keV and a 24-μm initial diameter to cover the 20-μm opening (entrance) of the collimator (Table 1, line (a)).

The TOPAS simulation has two steps for the microbeam collimation and cytoplasm irradiation, respectively. In the first step, a phase space scorer [10] was defined on the bottom surface of the water volume with a 24-μm diameter for covering the collimator exit. The phase space scorer is a built-in technique of TOPAS for saving and recalling the position, particle type, energy and momentum of a set of particles crossing a given surface. For simulating the transporting process of photons and electrons through the gold coaxial structure, the physics list *PENELOPE* was used, and the position and kinetic data of the extracted particles were saved in the phase space scorer at the collimator exit. In the second step, the phase space scorer with the recorded particle information was recalled as a virtual beam source (i.e. the phase space source), providing irradiation to the cytoplasm in the target cell. For simulating the subcellular dose-deposition process, the cytoplasm and nucleus volumes were defined as two dose scorers [10]. The physics list *g4em-dna* was used for the dose-deposition process, with the Auger process turned on. The range cutoff was adjusted from the default 50 μm to 0.1 μm. The lower energy limit *EMRangeMin* was set as 10 eV. The extracted cytoplasm and nucleus doses per unit incident fluence were both calculated in units of Gy/(10^5^μm^2^).

### Geometry adjustment

Based on the initial result of the starting model, the collimation geometry (length and inner/outer diameters) was adjusted to improve the hollow beam performance. The collimator length was extended from 1 mm to 10 mm (Table 1, line (b)), while the collimation diameters were evaluated by adjusting the inner diameter within the range of a fixed outer diameter of 20 μm, 50 μm and infinity. Here, the infinity of the outer diameter was evaluated using a finite 10-mm diameter, which is below the 20-mm size of the shielding shell but far larger than the 20-μm cell size. For the three fixed outer diameters, the incident beam size was also enlarged to cover the annular entrance of the collimator, from the initial diameter of 24 μm to one of 54 μm and then to 11 mm. For the 20-μm outer diameter, the inner diameter was adjusted from the initial 5 μm to 18 μm, which was slightly less than that of the outer diameter. For the 50 μm and infinity (10-mm) outer diameters, the inner diameter was increased from 5 μm to 25 μm, which exceeded the 20-μm cell diameter (Table 1, line (c–e)). Based on the geometry evaluations, a more practical collimation using a moderately increased 10-μm inner diameter, a 200-μm outer diameter and a fixed 1-mm length (Fig. 1b), with the incident beam source expanded to a 220-μm diameter (Table 1, line (f–k)), were used in the following simulations (see details in the result section ‘Influences and optimization of collimation geometry’).

### Evaluation of pretarget materials

After the geometry adjustment, a low-Z Mylar film was added to the practical model to evaluate the influence of a pretarget cell dish (Fig. 1c). The Mylar dish, with a thickness varying from 0 to 20 μm (Table 1, line (f, g)), was inserted between the collimator exit and the upper volume of water containing the target cell.

Considering that the beam collimation and cell irradiation were both performed in air, the pretarget air media must be evaluated for the influence on the microbeam performance. The simulations were performed on the model of the practical geometry (Table 1, line (f)), with the hollow cavity filled by air (Fig. 1c) and an air gap (1–10 μm) inserted between the collimator exit and the Mylar dish of a fixed 3-μm thickness (Table 1, line (h)). (More details in the result section ‘Pretarget materials: influences and practical settings’).

### Modeling of incident beam source

In addition to the material and geometry factors of the collimator and target dish, the influences of the energy and angular divergences of the incident beam were evaluated (Fig. 1d) using simulations for synchrotron and bremsstrahlung sources. For the synchrotron source, a Gaussian angular distribution (half-angle divergence) with a standard deviation of 0–1 mrad was used (Table 1, line (i)) instead of the monoenergetic parallel beam. For the bremsstrahlung source, the influence of energy divergence was first evaluated for a 5–100 kV source in the assumption of a parallel beam (Table 1, line (j)). The parallel source was set as a ‘continuous’ type in TOPAS, with the energy spectra calculated by SpekCalc 1.1 [15] for a Tungsten target. Subsequently, a Gaussian angular divergence (half-angle divergence) with a larger standard deviation of 0–15° was added to the 5–100 kV source to evaluate the combined influences of the energy and angular divergences (Table 1, line (k)). For simplicity, the potential correlation between the angular divergence and the photon energy (tube voltage) of a bremsstrahlung source was not considered. The beam source had the same divergence at each tube voltage.

## RESULTS

### Influence and optimization of collimation geometry

**Fig. 2:**
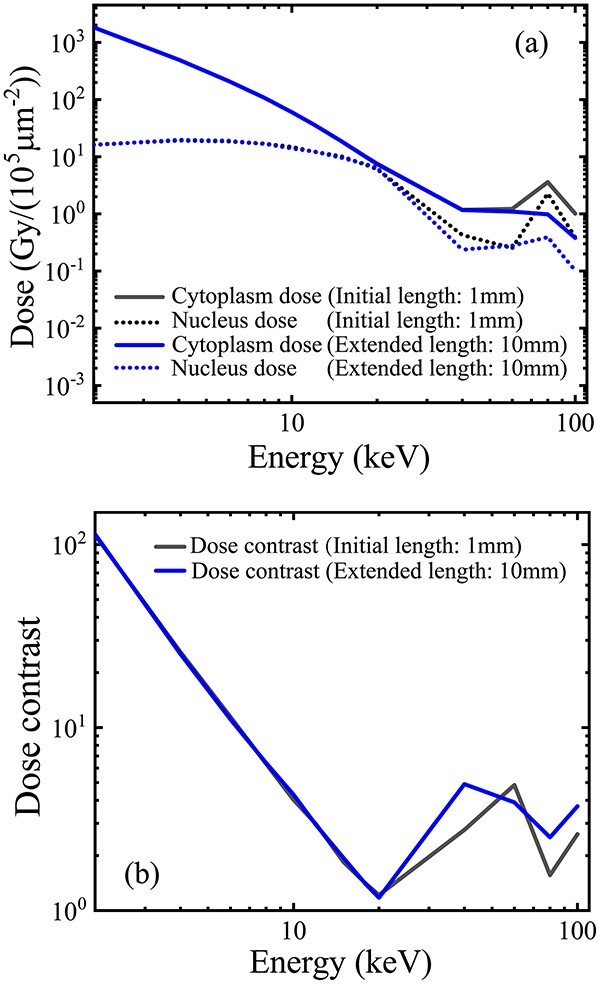
Microbeam performances of the initial model (1-mm length, 5-μm inner diameter and 20-μm outer diameter) (Table 1, line (a)) and comparison with the model of an extended length (10 mm) (Table 1, line (b)): (a) variation curves of the cytoplasm and nucleus doses and (b) variations of the dose contrast on the incident energy <100 keV. A monoenergetic parallel beam (1–100 keV), with a larger size (24 μm) to cover the entrance (20 μm) of the collimator, was used as the incident source. This simulation was performed in a vacuum.

The microbeam performance of the initial model is shown in Fig. 2. The cytoplasm dose, based on the extracted photons per unit incident fluence, and the nucleus dose, contributed by scattered lower-energy photons and electrons, both decrease with the increase in the incident beam energy but have different speeds (Fig. 2a). This caused a rapid decrease in the cytoplasm-to-nucleus dose contrast from 100 to 1 in the photon energy range < 20 keV (Fig. 2b). In the higher energy range from 20 to 100 keV, the dose contrast fluctuated but remained <10. Thus, within the range of incident energy <100 keV, the microbeam given by the initial collimation model was insufficient for high-contrast cytoplasm irradiation. Therefore, a geometry adjustment of the collimation length and inner/outer diameters was needed.

The collimation length was first adjusted from the initial 1 mm to a longer length of 10 mm, with the inner and outer diameters fixed to the initial sizes of 5 and 20 μm, respectively. However, the comparisons (Fig. 2a and b) indicate that the extension of the collimation length would achieve minimal improvement of the subcellular doses and the dose contrast. Therefore, the 1-mm collimation length was fixed in the following simulations.

**Fig. 3:**
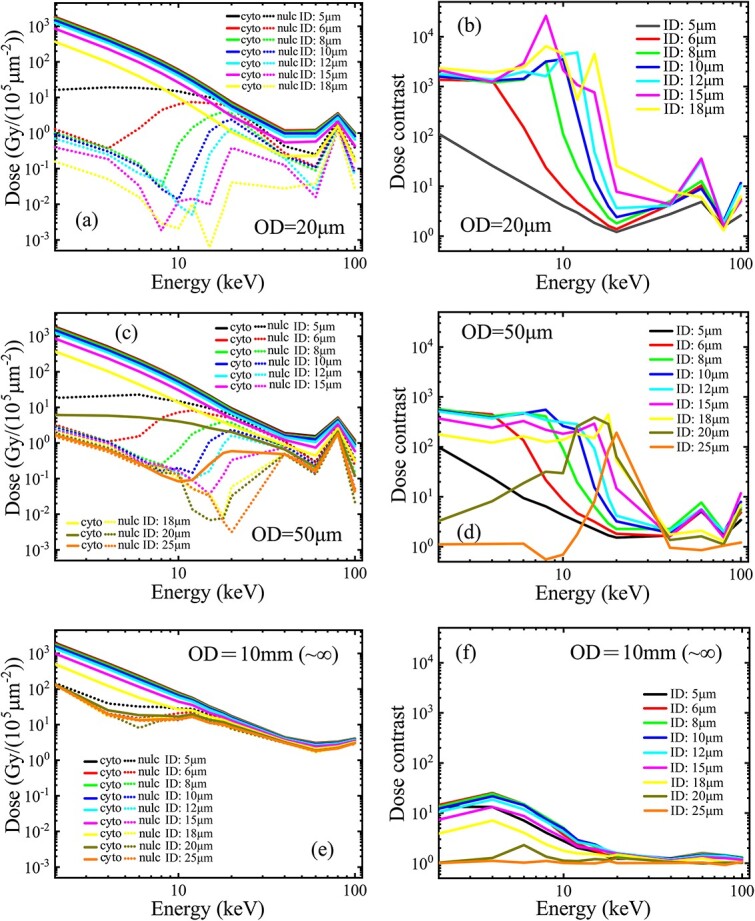
Microbeam performance evaluation on different inner and outer diameters (Table 1, line (c–e)): (a) the cytoplasm and nucleus doses and (b) the dose contrast on the incident energy for different inner diameters (5–18 μm) under the initial outer diameter (20 μm); (c) the subcellular doses and (d) the dose contrast for the different inner diameters (5–25 μm) under a fixed 50-μm outer diameter; (e) subcellular doses and (f) the dose contrast for the 5–25 μm inner diameters under a fixed 10-mm outer diameter (as infinity). The collimator length was fixed at 1 mm. A monoenergetic (1–100 keV) parallel beam, with an adapted size to cover the entrance of the collimator of different sizes, was used as the incident beam. Simulations were performed in a vacuum.

In the adjustment of the collimation diameters, the simulations (Fig. 3) indicate that for a fixed outer diameter of ≥20 μm cell size, an increase in the inner diameter from the initial 5 μm will always cause a simultaneous decrease in both the cytoplasm and nucleus doses in the energy range < 100 keV, but the faster drop of the nucleus dose (especially in the energy range < 20 keV) will lead to an increase of the dose contrast. Specifically, in the range of the incident energy <20 keV, the dose contrast can be enhanced up to 1000 (Fig. 3a and b) in the initial model with the 20-μm outer diameter (Table 1, line (c)) and to 200–500 (Fig. 3c and d) in the model with the 50-μm outer diameter (Table 1, line (d)); even in the model with an infinite outer diameter (i.e. 10 mm) (Table 1, line (e)), the dose contrast can still be enhanced to 5–10 (Fig. 3e and f). However, when both the outer and inner diameters exceed the 20-μm cell size, the dose contrast will decrease to 1 (Fig. 3d and f) because the entire cell has been totally shielded by the inner core.

However, for a fixed inner diameter above the 5-μm nucleus size but less than the 20-μm cell diameter, an increase in the outer diameter from 20 to 50 μm and to infinity (10 mm) (Table 1, line (c–e)) only causes a slight increase in the cytoplasm dose but a notable increase in the nucleus dose (Fig. 3a, c and e), leading to a monotonic decrease in the dose contrast with the increase in the outer diameter (Fig. 3b, d and f). Specifically, in the energy range < 20 keV, the dose contrast can be decreased from 1000 (Fig. 3b) in the initial model of 20-μm outer diameter down to 200–500 (Fig. 3d) in the model with a 50-μm outer diameter, and 5–10 (Fig. 3f) in the model of the infinite (10 mm) outer diameter.

Briefly, an increase in the inner diameter of the collimator creates a geometrical enhancement of the dose contrast, whereas an increase in the outer diameter causes a decrease in the dose contrast because of the faster increase in the nucleus dose. Moreover, although a moderate increase in the inner diameter can cause a dose-contrast enhancement through the faster decrease in the nucleus dose, the contrast enhancement occurs at the cost of cytoplasm dose decrease. For example, in both the models with 20- and 50-μm outer diameters, in the energy range of the incident beam <20 keV, an increase in the inner diameter to 6, 8, 10, 12 or 15μm led to a reduced cytoplasm dose of 98%, 90%, 80%, 70% and 46%, respectively, of the initial value under the 5-μm inner diameter.

Based on the evaluations, a practical collimation geometry (Table 1, line (f)) was adopted for the following models, with a fixed length of 1-mm, a moderately increased inner core diameter of 10-μm for contrast enhancement and an enlarged outer diameter of 200-μm in consideration of the machining difficulty for a high-aspect ratio hole with a micron-sized diameter but a millimeter-sized length (though at the cost of some decease in the dose contrast). A 200-μm diameter hole of the 1-mm length is relatively more achievable with current machining technologies.

**Fig. 4:**
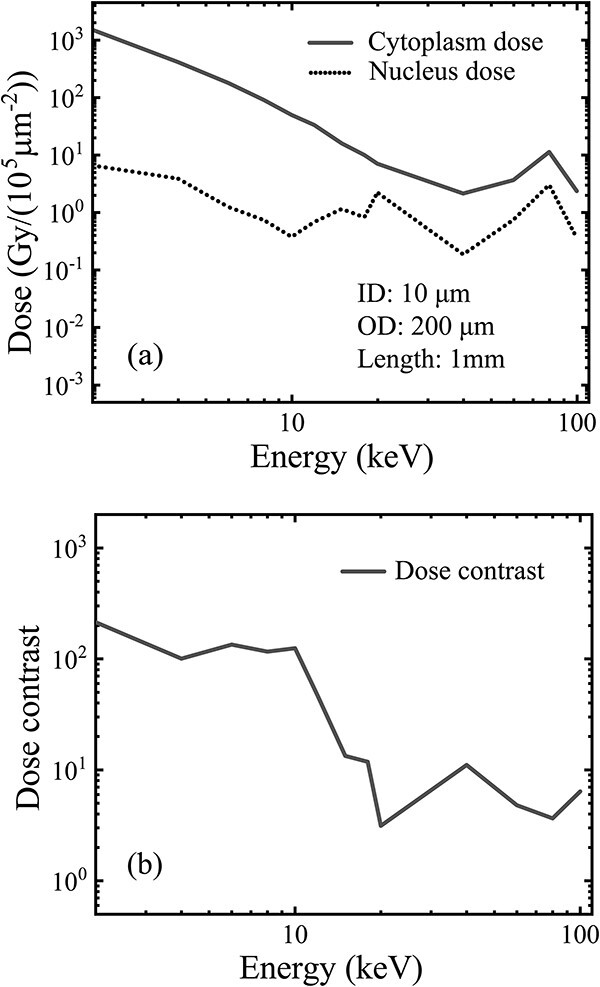
Performance of the microbeam extracted by a proposed practical collimation geometry (1-mm length, 10-μm inner diameter and 200-μm outer diameter): (a) cytoplasm and nucleus doses and (b) the dose contrast varying on the incident energy. A monoenergetic parallel beam (1–100 keV), with the beam size covering the collimator entrance, was used as the incident beam source. The simulation was performed in a vacuum.

For the practical geometry, the monoenergetic parallel incident beam was adapted to a 220-μm diameter for fully covering the collimator entrance. Other simulation settings were maintained the same as the starting model. As expected, such an enlarged outer diameter caused an increase in the nucleus dose and an insignificant increase in the cytoplasm dose (Fig. 4a), thus causing a decrease in the dose contrast from 1000 to 100 (Fig. 4b) compared with the curves under the initial 20-μm outer diameter (Fig. 3 and b), in the energy range < 20 keV. However, a dose contrast around 100 remains sufficient for practical use in the cytoplasm irradiation.

### Pretarget materials: Influences and practical settings

**Fig. 5:**
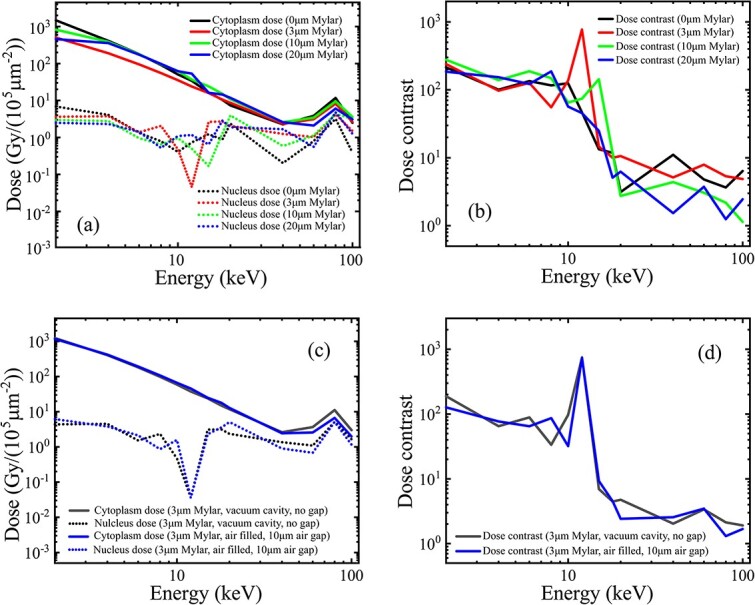
Influence of the Mylar dish and pretarget air under the practical geometry (1-mm length, 10-μm inner diameter and 200-μm outer diameter): (a) cytoplasm and nucleus doses and (b) the dose contrast varying on the incident beam energy for different (0–20 μm) thicknesses of Mylar dish; (c) doses and (d) the dose contrast for a fixed 3-μm Mylar dish, with and without the pretarget air (the air filled in the annular cavity and an air gap up to 10 μm between the collimator and the Mylar dish).

For the influence of the cell dish, the results (Fig. 5a and b) indicate that a Mylar dish of up to 20-μm thickness (Table 1, line (g)) caused insignificant decreases in both the cytoplasm and the nucleus doses, thus giving an approximately equivalent dose contrast in the energy range < 100 keV in comparison with the subcellular doses and the dose contrast (Fig. 4a and b) under the practical model of no dish (Table 1, line (f)). Notably, the calculated curves of the nucleus dose, and thus the dose contrast, fluctuated around the 10-keV incident energy because of the extremely low value near this energy, where an electron cutoff induced a drop of the nucleus dose that deposited from the exposed perinucleus area. In the following simulations, a 3-μm Mylar film, with sufficient strength and negligible absorption, was added to the model of the practical collimation geometry (Table 1, line (h)).

The influence of the predish air was also evaluated in the model of the practical geometry and 3-μm Mylar dish. The simulation results (Fig. 5c and d) indicate that the air in the annular cavity and a micron-thickness air gap up to 10 μm between the collimator exit and the bottom of the cell dish (Table 1, line (h)) both caused an insignificant difference in the variation curves from those obtained with the in-vacuum model (Fig. 4a and b). For a practical arrangement, the gap between the collimator and the cell dish is difficult to maintain to a consistent known thickness. To reduce the targeting error in cell irradiation, a minimized air gap is needed.

In the following simulations, the collimation geometry was updated to a coaxial structure of a 10-μm inner diameter, a 200-μm outer diameter and a 1-mm length. The annular hollow cavity was filled with air, while the exit of the collimator was closely attached to the bottom surface of the 3-μm Mylar dish, with a minimized (zero) air gap (Table 1, line (i–k)).

### Influences of incident beam characteristics

**Fig. 6:**
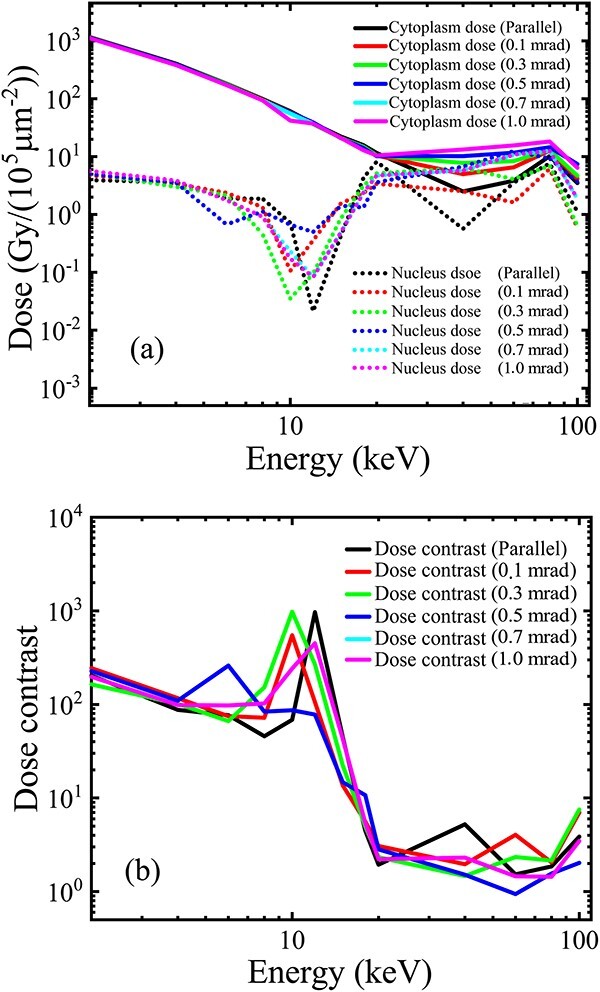
Influence of the beam divergence (a Gaussian angular distribution of a standard deviation in 0–1mrad) of a synchrotron source (1–100 keV), with the beam size covering the collimator entrance under the practical geometry (1-mm length, 10-μm inner diameter and 200-μm outer diameter): (a) cytoplasm and nucleus doses and (b) the dose contrast varying with the incident beam energy. The collimator annular cavity was filled with air. A 3-μm thick Mylar dish was inserted between the collimator and the water volume, with a minimized air gap.

For a synchrotron radiation source, the simulation results (Fig. 6) indicate that a Gaussian angular distribution of the beam divergence, with a 0.1–1 mrad standard deviation (Table 1, line (i)) causes minimal influence on the subcellular doses (Fig. 6a) and the dose contrast (Fig. 6b) compared with curves under the parallel source (Fig. 4a and b). Notably, the data fluctuation of the nucleus dose and the dose contrast near 10 keV incident energy still occurred because of the same technical reason detailed above.

**Fig. 7:**
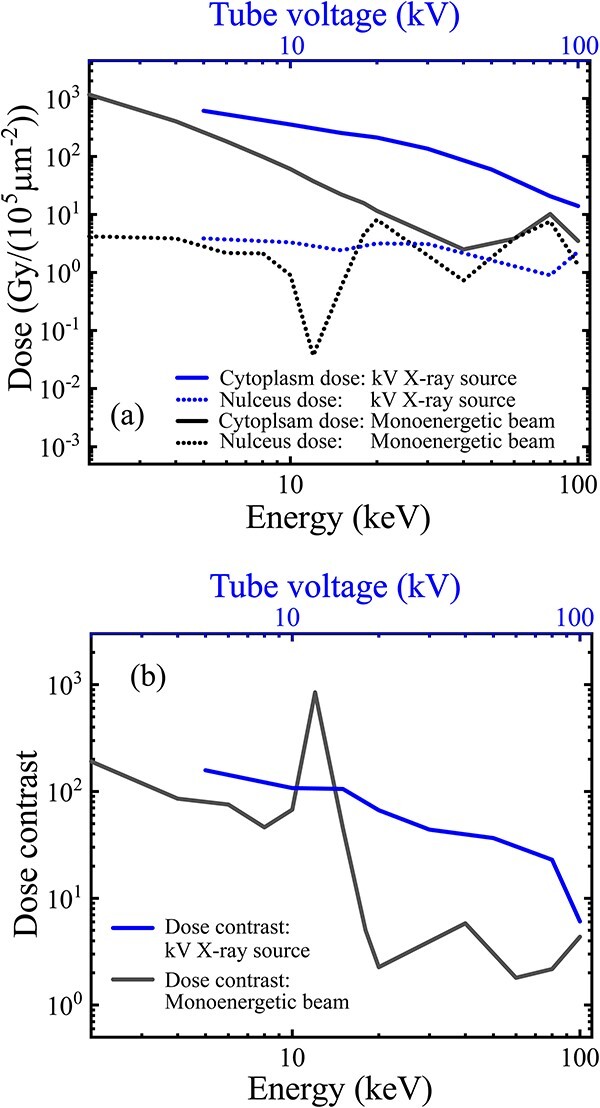
Independent influence of the energy divergence of bremsstrahlung source, assumed as a parallel beam covering the entrance of the collimator, with the energy spectra under a 5–100 kV tube voltage. The practical geometry (1-mm length, 10-μm inner diameter and 200-μm outer diameter) was used with the 3-μm Mylar dish and intracavity air. (a) Cytoplasm and nucleus doses and (b) the dose contrast varying on the tube voltage in comparison with the variation curves (Figs 4 and 5) obtained by a monoenergetic parallel beam (1–100 keV).

For a bremsstrahlung source, the independent influence of the energy spread was evaluated on the assumption of a parallel beam, with an energy spectrum of 5–100 kV tube voltage (Table 1, line (j)). The variation curves of the subcellular doses and dose contrast on the tube voltage are plotted in Fig. 7. Compared with the variation curves on the energy of the monoenergetic parallel beam (Fig. 5), the energy spread added to the parallel source causes a shift of the curve to the right in both the cytoplasm and nucleus doses (Fig. 7a), and consequently a curve shift to the right of the dose contrast (Fig. 7b). The curve shift of the doses and dose contrasts can be attributed to the energy spread of the bremsstrahlung source, which has a gradually decreasing portion of low-energy photons and a gradually increasing portion of high-energy photons with the increase in tube voltage.

For comparison in Fig. 7, the top axis of the tube voltage was aligned to the bottom axis of the mono energy by the equal value. Although the effective energy seemed more suitable, this was practically not a direct and explicit parameter of a bremsstrahlung source. For example, the effective energy of a 5–100-kV source is in a narrower range of 2.5–50 keV, compared with the monoenergy range of 5–100 keV. Thus, the comparison becomes ambiguous for the variation curves in different energy ranges. More importantly, using the variation curves on the tube voltage can provide a more intuitive result to show the usable range of the tube voltage that can support high-contrast cytoplasm radiation.

**Fig. 8:**
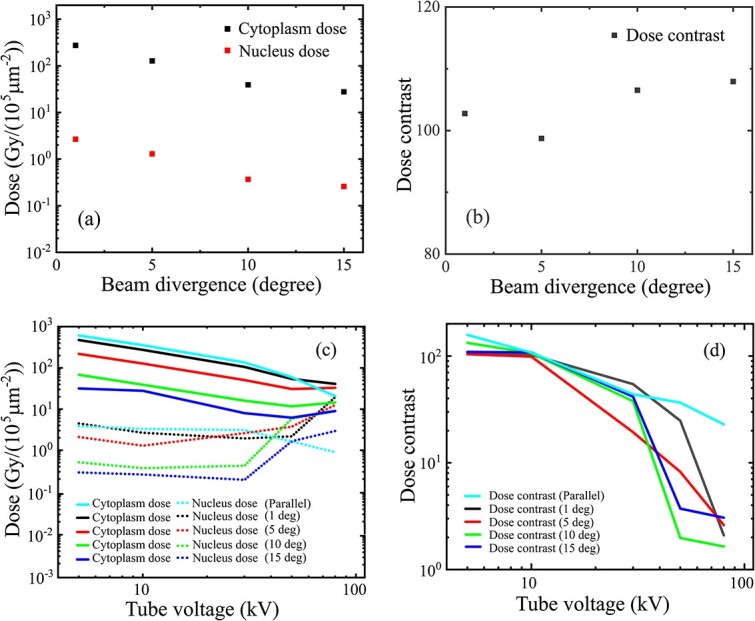
Combined influences of the energy spread and angular divergence of a bremsstrahlung source: (a) the cytoplasm and nucleus doses and (b) dose contrast variations on the beam divergence (0–15° of Gaussian half-angle divergence) under a fixed 10-kV tube voltage; (c) subcellular doses and (d) the dose contrast varying on the tube voltage (5–100 kV) for different beam divergence (0–15°). The practical geometry (10-μm inner diameter, 200-μm outer diameter and 1-mm length) was used with the 3-μm Mylar dish and intracavity air.

The combined influence of the energy spread and the angular divergence for a bremsstrahlung source was first evaluated with a fixed 10-kV voltage. The cytoplasm and nucleus doses (Fig. 8a) both decreased quasilinearly with the increase in the divergence, but the dose contrast maintained a quasiconstant value near 100 (Fig. 8b). At each tube voltage within the range < 100 kV (Table 1, line (k)), the subcellular doses had a similar quasilinear decrease (Fig. 8c) and quasiconstant contrast (Fig. 8d) with the increase in beam divergence.

It is also seen that with the increase in the tube voltage, the decreasing curve of the nucleus dose gradually transformed into an increasing curve (Fig. 8c) with the increase in the beam divergence, leading to a decrease in the divergence-independent dose contrast (Fig. 8d). The dose contrast curves (Fig. 8d) indicate an available range of the tube voltage up to 30 kV, under which a usable dose contrast around 50–100 can be acquired.

## DISCUSSION

To make a hollow X-ray microbeam for a cytoplasm irradiation based on the simulation results obtained in this study, several basic principles are proposed with explanations of the physical, material and geometrical arrangements for the collimator, target dish and incident beam source.

### General ideas

- Two key performances of the hollow X-ray microbeam are required, the extraction efficiency (cytoplasm dose per unit incident fluence) and the dose contrast (cytoplasm-to-nucleus ratio of dose). The extraction efficiency determines the dose rate in cytoplasm irradiation jointly with the fluence rate of the incident beam, whereas the dose contrast needs a value as high as possible to ensure so-called cytoplasm-only irradiation.
- To achieve these performances, a coaxial structure of a high-Z material such as gold can be employed for a hollow beam collimation, using the cylinder core for nucleus shielding, and the annular hollow cavity for guiding the incident photons to the target volume of cytoplasm in an adherent cell.

### Collimation geometry

- The collimation length (shielding core length) is determined by the required shielding level and the photon-attenuation ability of the employed material. For example, a 1-mm length of the gold shielding core ensures an attenuation down to 0.00005 of the incident photons <100 keV. However, further increasing the collimator length produces minimal improvement in microbeam performances (Fig. 2a and b).
- The initial inner and outer diameters of the collimator can be set as equal to the mean sizes of the nucleus and the entire cell, respectively (Fig. 1a). However, to improve the dose contrast in cell irradiation, both the initial diameters need further adjustment, i.e. increasing from the initial diameters as the lower limits.
- For a fixed outer diameter that is equal to or greater than the cell diameter, a moderate increase in the inner diameter from its lower limit (nucleus size) will decrease both the cytoplasm and the nucleus doses, but due to the faster drop of the nucleus dose, a useful increase in the dose contrast can be achieved (Fig. 3b and d), although this occurs at the cost of a decline of the extraction efficiency.
- The sharp drop of the nucleus dose around the incident energy 10–20 keV (Fig. 3a and c) with the increase in the inner diameter is due to the different geometry dependence from that of the cytoplasm dose. The cytoplasm dose is directly deposited by the extracted photons entering the cytoplasm volume through the annular hollow cavity. Therefore, only a slow decrease in the cytoplasm dose occurs because of the slow decrease in the exposed area of the cytoplasm with the increase in the inner diameter. However, the nucleus dose can only be deposited indirectly by the scattered photons and electrons from the exposed cytoplasm and peripheral area around the shielded nucleus. That is, only an indirect ‘marginal dose’ by the scattered electrons can be deposited from the side into the nucleus because of their limited range, together with another part of the nucleus dose by the scattered photons, which have an exponential decay (of no definite range). Therefore, the increased diameter of the inner shielding core that exceeds the nucleus diameter will impose a ‘cutoff’ effect on the lateral deposition of the scattered electrons into the nucleus. This indicates that an increased portion of lower energy electrons generated from the exposed perinucleus area, with a maximum range less than the radius of the inner shielding core, will be not able to reach the nucleus center, causing a fast drop in the nucleus dose due to the electron loss. Specifically, a specific radius of the inner core will give an upper limit to the incident energy, below which the range of secondary electrons is cut off by the inner core radius and cannot reach the nucleus center for a full cover, causing a decrease in the nucleus dose due to the appearance of a region of zero electron dose in the nucleus center. For the incident energy above the limit, the range of secondary electrons from the exposed perinucleus area is sufficient to reach the nucleus center for a full cover of the entire nucleus volume, thus giving a nucleus dose much closer to the cytoplasm dose. With the increase in the inner core radius, the incident energy subjected to the electron cutoff will be extended to a higher range (seen in Fig. 3a). However, because of the limitation in the inner core radius (not exceeding the cell radius and proposed to have a moderate increase compared with the nucleus radius), the maximum range of the incident energy subjected to the electron cutoff is also limited. In such an energy range of the electron cutoff, as the energy decreases, the range of the secondary electrons also decreases and the region of zero electron dose in the nucleus center will expand to the edge, giving a narrowing annular margin of the marginal dose, thus producing a decreasing value of the average electron dose in the nucleus volume. Finally, below a lower critical energy corresponding to the radius difference of the inner core and the nucleus, the electrons from the exposed perinucleus area will be totally cut off, giving a zero value of the electron dose in the nucleus volume. Such an electron dose cutoff will cause a sharp drop in the nucleus dose with the decrease of the incident energy. At this time, only the dose of scattered photons, which have no range limitation, remains to be deposited in the nucleus. The scattered photons entering the nucleus have a similar energy spectrum but a lower quantity (under per unit incident beam fluence) compared with the scattered photons produced in the exposed perinucleus area; therefore, the nucleus dose by the scattered photons will have a similar dependence on the incident energy (a higher value for lower energy, dominated by the photoelectric cross-section), although the curve is much lower than that of the cytoplasm dose. Briefly, with the incident energy decreasing below the critical energy, the fast drop in the nucleus dose by the electron cutoff and the slow increase in the rest dose of the scattered photons jointly create a downward peak in the curve of the nucleus dose around the critical energy and provide an energy range below this with an enhanced quasiconstant dose contrast, as shown in Fig. 3a and b.
- In terms of the practical geometry and size of target cells in this work, the 10-μm inner diameter exceeds the 5-μm nucleus by a 2.5-μm radius difference, which will give a critical energy to the incident beam of approximately 15keV (with a maximum continuous-slowing-down-approximation range of the secondary electrons of 5.1 μm [16]) for the cutoff occurrence on the secondary electrons that reach the nucleus center across the 5-μm distance, and a lower critical energy around 10 keV (with the maximum range of the secondary electrons of 2.5 μm [16]) for a full cutoff on the secondary electrons that reach the nucleus edge across the 2.5 μm radius difference. Therefore, a significant drop in the nucleus dose will appear with the incident energy decrease from 15 to 10 keV because of the electron cutoff by the 10-μm inner diameter. Below the incident energy of 10 keV, electrons from the perinucleus area are fully excluded, and the rest of the nucleus dose contributed by the scattered photons has a similar dependence but a much lower curve on the incident energy compared with that of the cytoplasm dose, giving an enhanced dose contrast of a quasiconstant value of approximately 1000, as shown in Fig. 3a and b.
- An excessive increase in the inner diameter that approaches or exceeds the target cell size will cause the dose contrast to decrease back to 1 (Fig. 3d) because at this time the cytoplasm and nucleus doses were both decreased to an equivalent low value (Fig. 3c) due to the overshielding by the oversized inner core.
- For a fixed inner core diameter between the diameters of the target nucleus and the target cell, an increase in the outer diameter from the lower limit (cell diameter) will cause a simultaneous increase in both the cytoplasm and nucleus doses by the scattered photons and electrons produced in the pericellular area exposed to the expanded beam field and deposited from the side into the cytoplasm and nucleus volumes. The dose contribution by the scattered photons can come from the whole pericellular area in the expanded beam field (with lateral attenuation but no range limitation), while the dose contribution by the secondary electrons can only be contributed from a portion of the pericellular area, where the distance to the cell edge (or nucleus edge) is below the maximum range of the secondary electrons under the incident energy. For example, in the incident energy range < 100 keV, a critical energy near 18 keV has a maximum range of the secondary electrons of approximately 7.5 μm, which is equal to the difference between the cell radius (10 μm) and the nucleus radius (2.5 μm). Therefore, below this incident energy, the dose contribution to the nucleus by the secondary electrons from the extended pericellular area is unavailable, and the increase in the nucleus dose is only given by the scattered photons. Conversely, the secondary electrons from the pericellular area can always be deposited into the adjacent cytoplasm, and therefore, for any incident energy, the increase in the cytoplasm dose includes both the contributions of the scattered photons and secondary electrons from the expanded pericellular area. Due to the higher dose contribution by secondary electrons to the cytoplasm, the increment of the cytoplasm dose seems to be slightly higher than that of the nucleus dose. However, with the increase in the outer diameter (thus exposing an enlarged pericellular area to the extended beam field), the relative increase in the nucleus dose is much higher relative to the cytoplasm dose, leading to a decrease in the dose contrast, especially in the incident energy range where the electron contribution to the nucleus dose is cut off (as discussed above). As an extreme case, when the outer diameter extends to an infinite size, the cytoplasm and nucleus doses will both approach the equilibrium values, whereas the dose contrast will approach its lower limit. However, in the low energy range of the incident beam, the dose difference produced by the electron cutoff still exists between the cytoplasm and the nucleus (as seen in Fig. 3e and f).
- Therefore, no adjustment of the collimation geometry (length, inner diameter, outer diameters) can achieve simultaneous improvements in both extraction efficiency and dose contrast. A practical geometry should first ensure a high enough dose contrast and then try to maintain a sufficient high extraction efficiency via a moderate increase in both the inner and outer diameters from the lower limits, respectively. Meanwhile, the capability of the current micromachining technologies should also be considered. In practice, high-Z material (e.g. gold) is recommended for the coaxial collimation structure. In this study, we propose a practical geometry (Table 1, line(f)) that proves sufficient in the cytoplasm irradiation use (Fig. 4a and b).

### Target arrangements

- A supporting dish for target cells is needed. The photon absorption through the cell dish is dependent on both the beam energy and the material and thickness of the cell dish. A low-Z film (like Mylar) of several-micron thicknesses can be used with a negligible absorption in the incident energy range < 100 keV.
- The air present in the annular cavity of the collimator and the micron-thickness air gap between the collimator and the target cell dish both have negligible influence on the microbeam performance. To minimize the pretarget scattering and lateral targeting error in the subcellular irradiation, a zero-air gap between the collimator exit and the bottom of the target dish is still required.

### Incident X-ray source

- Both synchrotron and bremsstrahlung radiation sources can use such a coaxial structure for collimation to obtain a hollow X-ray microbeam for cytoplasm irradiation.
- The divergence of a typical synchrotron radiation source (Table 1, line (i)) causes negligible influence on the performance of the microbeam (Fig. 6a and b) compared with that of a monoenergetic parallel beam source.
- In comparison with the variations under a monoenergetic source, the subcellular doses and dose contrast under a bremsstrahlung source present a curve shift (slower decline) on the tube voltage because of the energy divergence. The descent curve of the dose contrast indicates an available range of the tube voltage up to 30 kV for a bremsstrahlung source to extract a high-contrast hollow X-ray microbeam (Fig. 7a and b).
- The angular divergence of a bremsstrahlung source causes a simultaneous decrease in both the cytoplasm and the nucleus doses (Fig. 8c) but maintains a divergence-independent curve of the dose contrast that only decreases with the increase of the tube voltage (Fig. 8d). To ensure a sufficient extraction efficiency of the microbeam for a bremsstrahlung beam source, an incident divergence as small as possible is required.

### A comparative evaluation

Using the simulation method, a comparative evaluation can also be made on the performance of the reported hollow X-ray microbeam by the Photon Factory [9]. For the collimation geometry and material arrangements (a gold post of 15-μm diameter and 20-μm thickness for nucleus shielding on a Si_3_N_4_ film of 100-μm thickness) under a 5.35-keV synchrotron X-ray within a 300 × 300-μm beam field as the incident source, the cytoplasm irradiation to adherent V79 cells (modeled as of a 10-μm thickness, a mean area of 168 μm^2^ of the nucleus and a 360 μm^2^ area of the cell) on a 3-μm–thick PP dish can achieve a cytoplasm-to-nucleus dose contrast of approximately 80.

## CONCLUSION

The simulation study described herein demonstrates that the use of high-Z coaxial collimation enables the technical achievement of a hollow X-ray microbeam for high-contrast cytoplasm irradiation. The simulation results indicated that the collimator geometry, pretarget media and incident beam characteristics are essential factors determining the microbeam performance. The collimation length is determined by the energy range of the incident beam and the shielding level requirement, while the inner and outer diameters need to be carefully increased from their lower limits (nucleus and cell diameters) to achieve a sufficient dose contrast and maintain a high enough extraction efficiency. To minimize the targeting error in cell irradiation, a minimized air gap is proposed between the collimator exit and the target cell dish. Based on the principles of collimation geometry and source-target arrangement, a gold collimator with a proposed geometry (10-μm inner diameter, 200-μm outer diameter and 1-mm length) is recommended for practical use. Simulations with this gold collimator demonstrate that synchrotron and bremsstrahlung radiation sources can be used to achieve a hollow X-ray microbeam for cytoplasm irradiation. For a synchrotron source with the energy <10 keV, a dose contrast of approximately 100 can be achieved, while for a bremsstrahlung source with the tube voltage <30 kV, a dose contrast of approximately 50–100 can be acquired.

## CONFLICT OF INTEREST

None.

## Funding

This work was supported by the National Natural Science Foundation of China (No. 12075063, 12275056).

## Ethics approval and consent to participate

None.

## Consent for publication

All the authors agreed to be published.

## Data availability

The datasets used and/or analyzed during the current study are available from the corresponding author on reasonable request.

## Presentation at a conference

No.

## References

[1] Folkard M, Schettino G, Vojnovic B et al. A focused ultrasoft x-ray microbeam for targeting cells individually with submicrometer accuracy. Radiat Res 2001;156:796–804. 11741504 10.1667/0033-7587(2001)156[0796:afuxrm]2.0.co;2

[2] Schettino G, Folkard M, Prise KM et al. Low-dose studies of bystander cell killing with targeted soft X rays. Radiat Res 2003;160:505–11. 14565833 10.1667/rr3060

[3] Ghita M, Fernandez-Palomo C, Fukunaga H et al. Microbeam evolution: from single cell irradiation to pre-clinical studies. Int J Radiat Biol 2018;94:708–18. 29309203 10.1080/09553002.2018.1425807

[4] Tomita M, Kobayashi K, Maeda M. Microbeam studies of soft X-ray induced bystander cell killing using microbeam X-ray cell irradiation system at CRIEPI. J Radiat Res 2012;53:482–8. 22510578

[5] Suzuki M, Funayama T, Suzuki M et al. Radiation-quality-dependent bystander cellular effects induced by heavy-ion microbeams through different pathways. J Radiat Res 2023;64:824–32. 37658690 10.1093/jrr/rrad059 PMC10516730

[6] Choi J, Kim G, Cho SB et al. Radiosensitizing high-Z metal nanoparticles for enhanced radiotherapy of glioblastoma multiforme. J Nanobiotechnol 2020;18:122. 10.1186/s12951-020-00684-5 PMC7470617 32883290

[7] Chithrani DB . Intracellular uptake, transport, and processing of gold nanostructures. Mol Membr Biol 2010;27:299–311. 20929337 10.3109/09687688.2010.507787

[8] Yokoya A, Usami N. Targeting specific sites in biological systems with synchrotron X-ray microbeams for radiobiological studies at the photon factory. Quantum Beam Sci 2020;4:2. 10.3390/qubs4010002.

[9] Maeda M, Tomita M, Maeda M et al. Exposure of the cytoplasm to low-dose X-rays modifies ataxia telangiectasia mutated-mediated DNA damage responses. Sci Rep 2021;11:13113. 34219128 10.1038/s41598-021-92213-z PMC8255317

[10] Perl J, Shin J, Schumann J et al. TOPAS: an innovative proton Monte Carlo platform for research and clinical applications. Med Phys 2012;39:6818–37. 23127075 10.1118/1.4758060 PMC3493036

[11] Faddegon B, Ramos-Méndez J, Schuemann J et al. The TOPAS tool for particle simulation, a Monte Carlo simulation tool for physics, biology and clinical research. Phys Med 2020;72:114–21. 32247964 10.1016/j.ejmp.2020.03.019 PMC7192305

[12] Alberts B, Johnson A, Lewis J et al. Molecular Biology of the Cell, 6th edn. New York: Garland Science, 2015, 179.

[13] Tracy BL, Stevens DL, Goodhead DT et al. Variation in RBE for survival of V79-4 cells as a function of alpha-particle (helium ion) energy. Radiat Res 2015;184:33–45. 26121227 10.1667/RR13835.1

[14] Arzumanian VA, Kiseleva OI, Poverennaya EV. The curious case of the HepG2 cell line: 40 years of expertise. Int J Mol Sci 2021;22:13135. 34884942 10.3390/ijms222313135 PMC8658661

[15] Poludniowski G, Landry G, DeBlois F et al. SpekCalc: a program to calculate photon spectra from tungsten anode x-ray tubes. Phys Med Biol 2009;54:N433–8. 19724100 10.1088/0031-9155/54/19/N01

[16] Walters R . Stopping-power & range tables for electrons, protons, and helium ions. NIST Stand Ref Database 2017;124. 10.18434/T4NC7P (Last Update: July 2017).

